# Supplementary material for: The bZIP Transcription Factor GmbZIP15 Negatively Regulates Salt- and Drought-Stress Responses in Soybean
Source: Int J Mol Sci. 2020 Oct 21;21(20):7778. doi: 10.3390/ijms21207778 (PMC7589023; doi:10.3390/ijms21207778)
Supplement: Supplementary file 1 [file ijms-21-07778-s001.zip › supplementary material/supplementary figure.pdf]

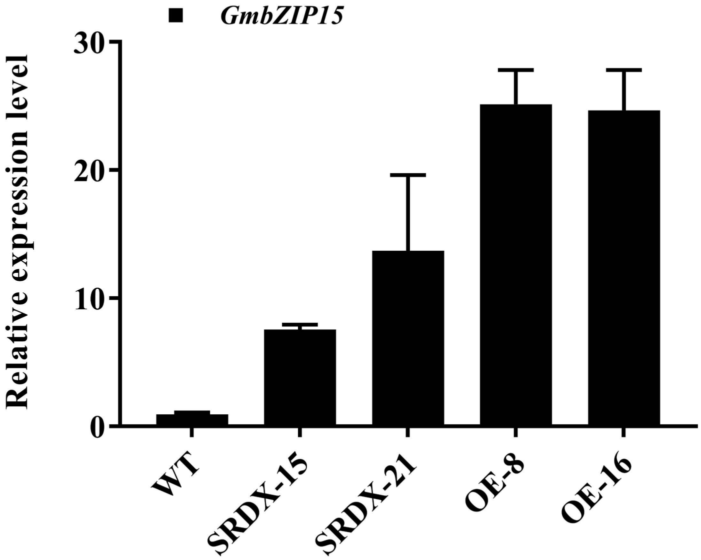

Figure S1. Transcript levels of *GmbZIP15* in transgenic soybean plants. Errors bars indicate ± SD of three biological replicates. Significant differences were determined by one-way ANOVA, P < 0.05.

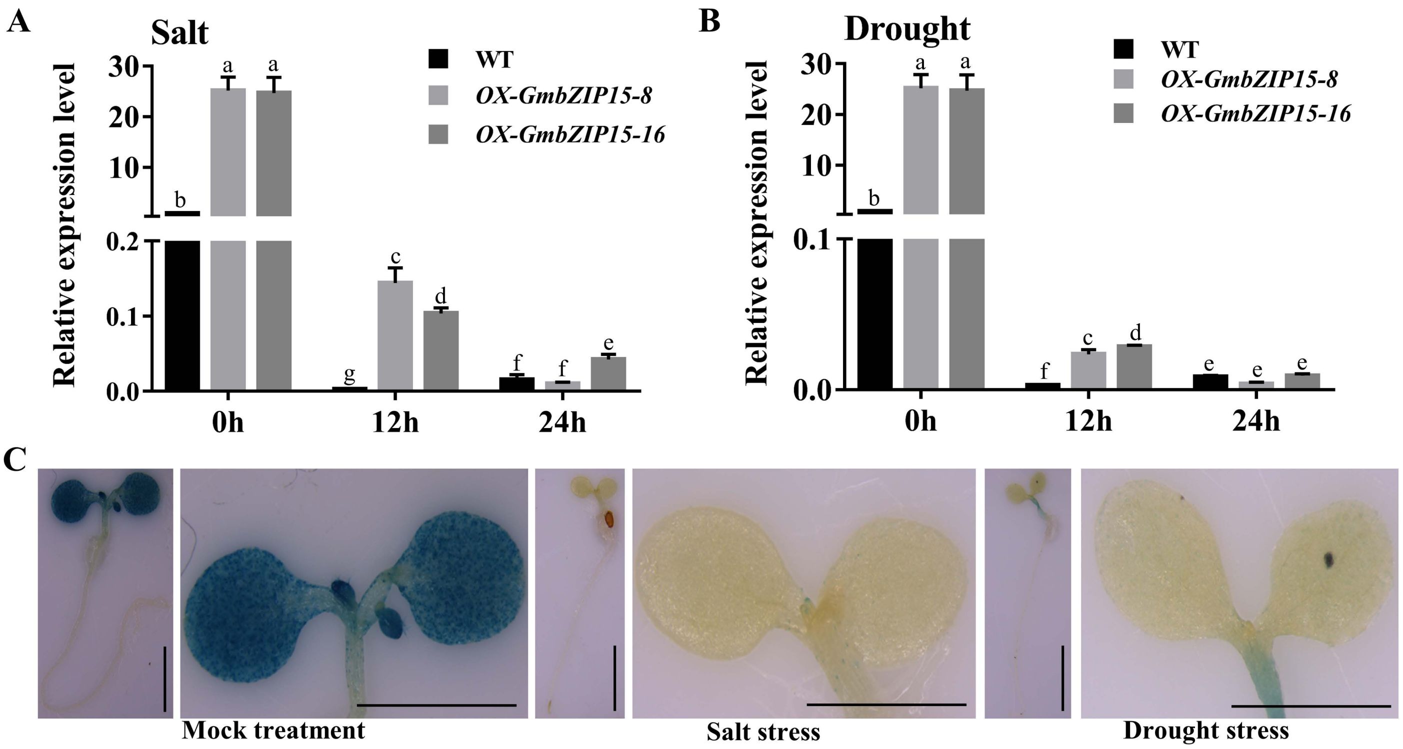

Figure S2. Expression pattern of *GmbZIP15* under salt and drought stress condition. (A-B) Relative expression level of GmbZIP15 in response to salt (A) and drought (B) stress. Two-week-old WT and *OX-GmbZIP15-8/16* soybean seedlings were treated with 200mM NaCl or 300mM mannitol for 0, 6, 12, and 24 h, and the expression of *GmbZIP15* was detected by qRT-PCR. (C) The expression pattern of *GmbZIP15* by GUS staining of *pGmbZIP15::GUS* transgenic seedlings under mock and stress conditions. At least 15 seedlings from each sample were used for every technical replicate and three biological replicates were conducted. The seeds were germinated on ½ MS medium supplemented with 100mM NaCl or 200 mM mannitol for one week. Bar= 1mm. Errors bars indicate ± SD of three biological replicates. Significant differences between samples labeled with different Roman (a, b, c) were determined by one-way ANOVA, $P < 0.05$.

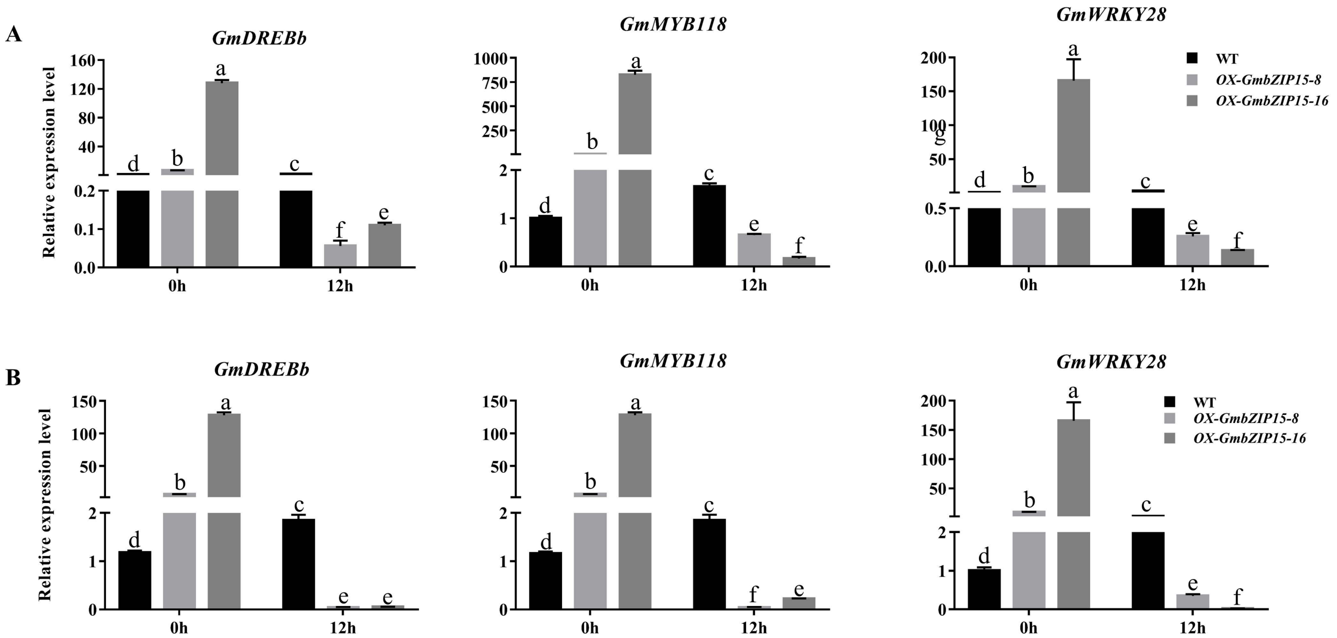

Figure S3. Transcript levels of stress-responsive genes in WT and *GmbZIP15* overexpression soybean plants in response to osmotic stress. Time courses of relative transcript levels of *GmDREBb*, *GmMYB118* and *GmWRKY28* in WT and *OX-GmbZIP15-8/16* lines treated with 200mM NaCl(A) or 300mM mannitol(B). Errors bars indicate ± SD of three biological replicates. Significant differences between samples labeled with different Roman (a, b, c) were determined by one-way ANOVA, $P < 0.05$.

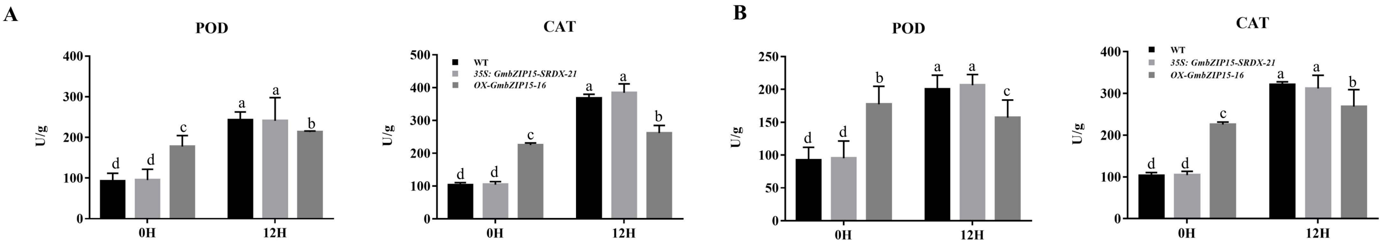

Figure S4. Antioxidants activity of WT and *GmbZIP15* transgenic soybean plants in response to osmotic stress. The POD and CAT activities of WT and transgenic soybean plants were measured after 200mM NaCl treatment for 24 h (A) and 300mM mannitol treatment for 24 h (B). Errors bars indicate ± SD of three biological replicates. Significant differences between samples labeled with different Roman (a, b, c) were determined by one-way ANOVA, $P < 0.05$.

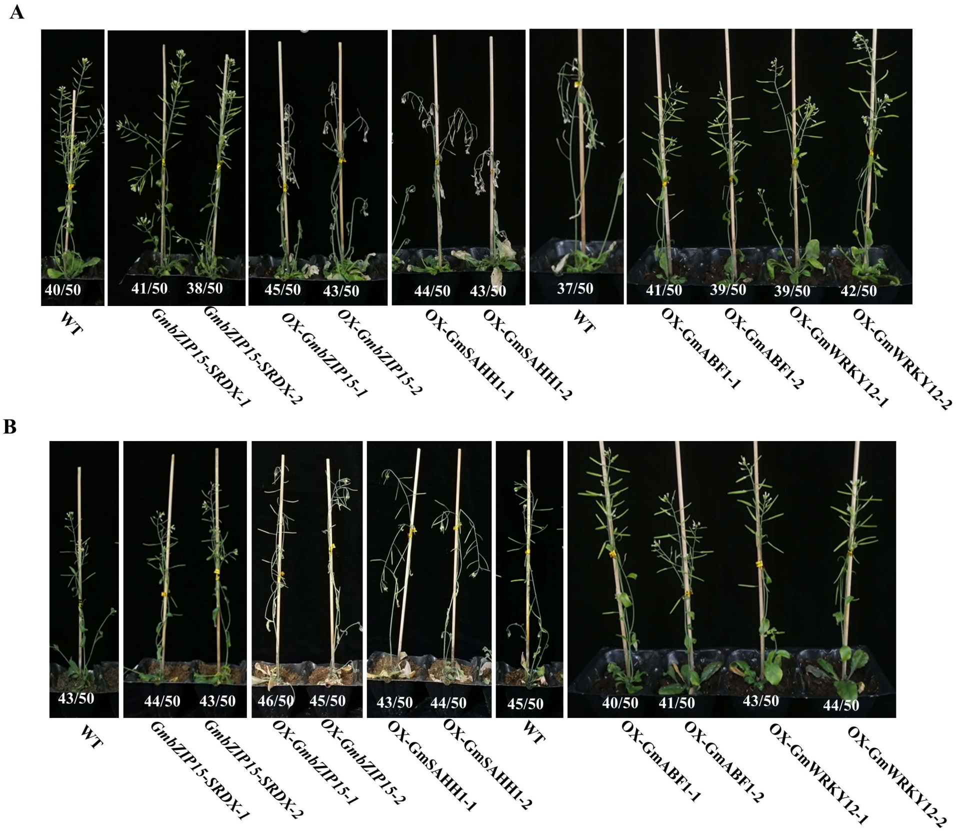

Figure S5. Phenotype analysis of WT, *35S:GmbZIP15-SRDX*, *OX-GmbZIP15*, *OX-GmSAHH1*, *OX-GmWRKY12* and *OX-GmABF1* transgenic Arabidopsis seedlings in response to osmotic stress at seedling stage. (A) For salt stress, five-week old WT and transgenic Arabidopsis seedlings were watered with 150mM NaCl for two weeks. (B) For drought stress, five-week old WT and transgenic Arabidopsis seedlings were withheld water for two weeks. Numbers in the panels denote the frequencies of the phenotypes shown.

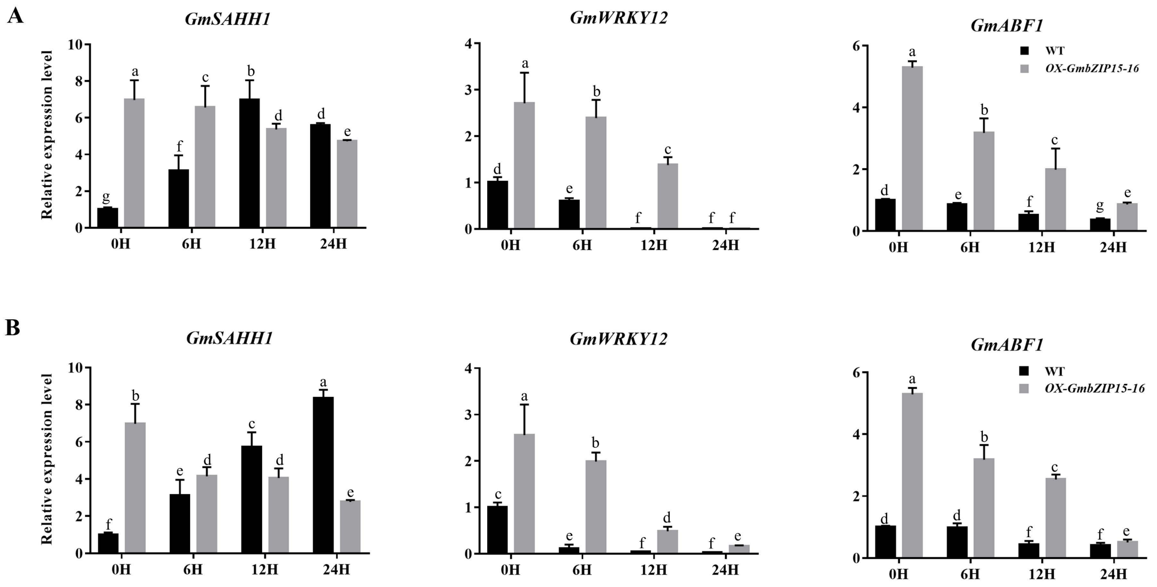

Figure S6. Expression pattern of *GmSAHH1*, *GmWRKY12* and *GmABF1*in response to osmotic stress. Expression level of *GmSAHH1*, *GmWRKY12* and *GmABF1* in WT and *OX-GmbZIP15-16* transgenic soybean plants after 150mM NaCl (A) or 300mM mannitol (B) treatment.
